# Prepare-and-measure and entanglement simulation beyond qubits

**DOI:** 10.1038/s41598-026-51465-3

**Published:** 2026-05-09

**Authors:** Mani Zartab, Giulio Gasbarri, Gael Sentís, Ramon Muñoz-Tapia

**Affiliations:** 1 Física Teòrica: Informació i Fenòmens Quàntics, Departament de Física, Universitat Autònoma de Barcelona, Bellaterra, 08193 Barcelona, Spain; 2 Naturwissenschaftlich-Technische Fakultät, Universität Siegen, 57068 Siegen, Germany; 3 Ideaded, Carrer de la Tecnologia, 35, Viladecans, 08840 Barcelona, Spain

**Keywords:** Mathematics and computing, Physics

## Abstract

For two non-communicating parties, quantum theory can give rise to probability distributions of outcomes that no local classical model can reproduce without communication. However, in the case of two-dimensional systems ($d=2$), it is known that allowing a finite amount of classical communication in addition to shared classical resources makes it possible to simulate these quantum correlations. Whether such a simulation remains possible in higher dimensions is still an open question. In this work, we identify the key features of the exact classical protocol in $d=2$, and use them to construct robust approximate protocols in higher dimensions. We assess their performance through a randomized numerical study based on the Total Variation Distance. Our approach exactly reproduces the quantum probability distributions for $d=2$, and performs very well compared to existing protocols for higher dimensions, being the most robust protocol in all cases studied. These results offer new insights into the analytical structure of classical protocols in higher dimensions.

## Introduction

Since Bell’s pioneering work^1–4^, it has been well established that in certain quantum scenarios, two non-communicating, spatially separated parties, say Alice and Bob, can perform independent measurements on shared systems and observe correlations that no classical model can statistically reproduce. This phenomenon has profound implications for quantum information science, enabling quantum advantages in tasks such as device-independent cryptography^5^ and randomness generation^6^, and can therefore be regarded as a resource. Consequently, understanding and quantifying this statistical observation—so-called non-locality—is of both foundational and practical importance.

One approach to tackle the quantification of this resource is through communication complexity, which links quantum correlations to classical communication requirements^7^. In this framework, the difficulty of classically simulating quantum correlations is measured by the additional amount of information that must be exchanged between two spatially separated parties to reproduce quantum statistics. In other words, if finite communication is permitted, can a classical model (using solely classical resources) reproduce correlations resulting from a scenario where quantum resources are available? This perspective not only deepens our understanding of the fundamental limits of classical and quantum theories but also has implications for computational complexity and distributed computing^7,8^.

In this context, Toner and Bacon made a significant contribution by proposing a protocol, using a single bit of classical communication, plus a classical shared resource—referred to as *shared randomness*—to exactly reproduce the correlations observed in local projective measurements on a shared maximally entangled state–a setting we refer to as the *entanglement scenario*^9^. They further demonstrated that in the closely related *prepare-and-measure* (PM) *scenario*, where Alice prepares a state and transmits it to Bob, who then performs a measurement, the quantum statistics of projective measurements on qubits can be reproduced by communicating just two classical bits. More recently, Renner, Tavakoli, and Quintino extended these results to general measurements, i.e, Positive Operator-Valued Measures (POVMs), proposing a protocol that maintains the same communication cost^10^. A key result of their work is the proof that two bits of communication are both necessary and sufficient for the task. These works focus on the defined scenarios, in $d=2$.

In higher-dimensional systems $(d \ge 3)$, the challenge of classically simulating the same quantum scenarios becomes more demanding, and extending these results to higher dimensions remains open. In particular, it is still an open question whether classical models, even when augmented with any finite amount of communication, can exactly replicate quantum statistics in these settings.

Nevertheless, motivated by this challenge, several classical models have been proposed that approximate quantum statistics in higher dimensions. For instance, by using a standard sampling tool in statistics, i.e., the rejection method^11,12^, we have designed two protocols of communication for simulating the PM scenario, based on two ontological models introduced by Rudolph in^13^, which we refer to as PRUD-1 and PRUD-2. Additionally, Montina proposed three protocols in^14^—PMON-1, PMON-2A, and PMON-2B—for the entanglement scenario, extending previous models to dimensions higher than two $d>2$. Notably, PRUD-1, PMON-1 and PMON-2A are exact for $d=2$. Moreover, protocols designed for the PM scenario can be extended to the entanglement scenario, and vice versa, via the Choi–Jamiołkowski isomorphism^14–16^.

In this context, two notable gaps remain in the current literature. First, the protocols developed for the case of $d=2$^9,10,17–20^ rely heavily on geometric arguments to show consistency with quantum mechanical predictions, but they do not identify the physical principles that account for their success. Secondly, despite offering valuable insights into potential exact strategies, to the best of our knowledge, the performance of the approximate protocols has not been assessed through explicit, randomized numerical studies for both scenarios. Given that these models are inherently approximate for $d\ge 3$, it is crucial to evaluate how closely they replicate quantum theory using rigorous distance metrics, such as Kullback–Leibler Divergence (KLD) or Total Variation Distance (TVD). As justified in the Results section, we utilize the TVD due to its operational suitability for the purposes of this study.

This work offers a new perspective on the classical simulations for $d=2$ presented in^9,10,17,18,20^, and exploits it to construct a probability distribution defined over *SU*(*d*) that yields a novel approximate protocol for simulating projective measurements for $d>2$. The protocol is highly accurate, and the main new theoretical ingredients that our analysis identifies can play an essential role in building an exact protocol for higher dimensions. Additionally, we conduct a numerical study to compare the performance of our protocol with that of other approximate protocols. Addressing these gaps is essential for advancing our understanding of classical simulations of quantum systems in higher dimensions.

The numerical assessment is performed for both randomized and structured input setups, considering $d=2,3,4$, and measured by the TVD for both the PM and entanglement scenarios. For the randomized cases, our analysis involves $n=100$ input setups, each with an output sample size of order $N_\text {out}\approx 10^{5}$. We show that our protocol exactly reproduces quantum statistics for $d=2$, and consistently ranks among the best-performing models for $d=3$ and $d=4$. In these higher dimensions, it achieves the highest mean accuracy for $d=3$ for both scenarios, and its performance is statistically on par with the other leading protocols, within the statistical error of the mean from the numerical simulations. Moreover, the two additional non-random cases considered reveal our protocol is the most robust one across all cases, by which we mean that it maintains consistently low TVD—and hence close agreement with the target quantum statistics—across both randomized and structured setups, without exhibiting a significant increase of the TVD relative to the protocols already available in the literature.

The structure of this paper is as follows: In the Preliminaries subsection, we provide a theoretical foundation on which the work is established. In the Methods section, we define the PM scenario and entanglement scenario and present our method for generalizing from the exact protocol of $d=2$ to $d\ge 3$. The Results section provides numerical results and a comparative analysis of our protocol with existing ones. In the Discussion and Conclusions section, we conclude by discussing key challenges and proposing directions for future research. The detailed numerical results are reported in the Supplementary Material (SM).

### Preliminaries

In this subsection, we introduce the main concepts and notation used throughout the paper, with emphasis on Bell non-locality, shared randomness, and classical communication-assisted simulation models.

Consider a bipartite conditional probability distribution $\textrm{P}(a,b | \mathscr {A},\mathscr {B})$, where $\mathscr {A}$ and $\mathscr {B}$ denote the inputs (measurement settings) chosen by Alice and Bob, and *a* and *b* denote the corresponding outputs.

A *local hidden-variable* (LHV) model assumes that the correlations are mediated by a shared classical variable $\lambda$, sampled from a probability distribution $\rho (\lambda )$ on a hidden-variable space $\Lambda$. Under the assumptions of *measurement independence* and *Bell locality*, the distribution admits the decomposition

1 $$\begin{aligned} \textrm{P}_{\textrm{LHV}}(a,b| \mathscr {A},\mathscr {B}) = \int _{\Lambda } d\lambda \rho (\lambda ) \textrm{P}_\textrm{A}(a |\mathscr {A},\lambda )\,\textrm{P}_\textrm{B}(b | \mathscr {B},\lambda ). \end{aligned}$$

Here, *measurement independence* means that the hidden variable is statistically independent of the inputs,

2 $$\begin{aligned} \rho (\lambda |\mathscr {A},\mathscr {B})d\lambda =\rho (\lambda )d\lambda , \end{aligned}$$

and *Bell locality* means that, conditioned on $\lambda$, each party’s response depends only on the local input. Equivalently, $\mathrm {P_A}(a| \mathscr {A},\lambda )$ is independent of $\mathscr {B}$ and *b*, and $\mathrm {P_B}(b| \mathscr {B},\lambda )$ is independent of $\mathscr {A}$ and *a*. This is the locality assumption used in Bell’s theorem ^1^.

The variable $\lambda$ is common to both parties and may vary between repetitions of the protocol. It therefore represents a pre-established classical resource shared by Alice and Bob, *i.e.*, *shared randomness*.

Throughout this work, by *non-locality* we mean *Bell non-locality*: the impossibility of representing $\textrm{P}(a,b| \mathscr {A},\mathscr {B})$ in the Bell-local form of Eq. (1). Bell’s theorem shows that there exist quantum correlations $\mathrm {P_Q}(a,b| \mathscr {A},\mathscr {B})$ that do not admit such a decomposition ^1–4^. In finite-input/output scenarios, this is equivalently witnessed by the violation of a Bell inequality (e.g., a CHSH-type inequality) ^4^.

A standard operational way to quantify Bell non-locality is to ask how much *classical communication* must be added to shared randomness in order to reproduce a given set of quantum correlations. In a one-way communication-assisted model, Alice sends a classical message *C* to Bob. For the one-bit case, where *C* takes the values $c\in \{0,1\}$, we define

3 $$\begin{aligned} \textrm{P}_{\textrm{LHVC}}(a,b|\mathscr {A},\mathscr {B})= \int _{\Lambda } d\lambda \rho (\lambda ) \sum _{c=0}^{1}\textrm{P}_{\textrm{A},C}(a,c|\mathscr {A},\lambda ) \textrm{P}_\textrm{B}(b|c,\mathscr {B},\lambda ). \end{aligned}$$

This model is strictly more general than Eq. (1), because Bob’s response may now depend explicitly on the communicated bit *c*, in addition to $\mathscr {B}$ and $\lambda$. If, for a given quantum scenario, one can construct a model of the form (3) such that

4 $$\begin{aligned} \textrm{P}_{\textrm{LHVC}}(a,b | \mathscr {A},\mathscr {B})=\mathrm {P_Q}(a,b | \mathscr {A},\mathscr {B}), \qquad \end{aligned}$$

for all $\mathscr {A},\mathscr {B},a,b,$ then the protocol provides an exact classical simulation of the quantum statistics.

The minimum amount of communication required in such a simulation provides an operational quantifier of Bell non-locality. A canonical example is the Toner-Bacon protocol^9^, which exactly simulates the correlations of local projective measurements on a maximally entangled two-qubit state using shared randomness plus one classical bit.

The same formal framework extends beyond Bell scenarios, including PM scenarios. In all such cases, the concrete realization of the shared randomness $\lambda$ is protocol-dependent, but its role is always the same: $\lambda$ is a classical resource sampled before the inputs are chosen and shared by the parties. For example, $\lambda$ represents a pair of independently and uniformly distributed three-dimensional real vectors in the protocol provided in^9,10^; in PRUD-1, it consists of rank-1 projectors of dimension $d\ge 3$ weighted similarly to the case in Kochen and Specker’s model^21^; In PRUD-2, the problem is embedded in $(d+1)$-dimensional space. Again, the shared randomness is defined via rank-1 projectors, but with a different distribution; PMON-2A and PMON-2B similarly extend Kochen and Specker’s model, only differing in the nature of shared randomness: PMON-2A employs the classical description of randomly sampled pure states, while PMON-2B utilizes the classical description of random bases, similar to how we define shared randomness in our design.

## Methods

In what follows, we introduce both the PM and entanglement frameworks and present our classical communication strategy for simulating them.

### PM scenario

The PM scenario is defined as follows: Alice prepares a general quantum state of dimension *d*: $\sigma _\textrm{A} \in \mathscr {L}(\mathscr {H}_\textrm{A})$. She sends this state to Bob, who performs a general measurement and reports the outcome *b*, chosen from *m* possible values. Such measurements are formally described by a POVM, denoted by $\mathscr {B}=\{{B_0,B_1,\cdots ,B_{m-1}}\}$, where each operator $B_i$ is positive semi-definite and collectively satisfies the completeness relation^22^

5 $$\begin{aligned} \sum _{i=0}^{m-1} B_i\ = I. \end{aligned}$$

If each operator also fulfills the additional constraint $B_i^2 =B_i$, the measurement becomes a projective one, implying the number of outcomes matches the dimension of the Hilbert space, i.e., $m=d$. Independent of the measurement type, the probability of observing outcome *b* given the quantum state $\sigma _\textrm{A}$, is determined by Born’s rule:

6 $$\begin{aligned} \textrm{P}_{\textrm{Q}}(b|\sigma _\textrm{A},\mathscr {B})=\text {Tr}[B_b\sigma _\textrm{A}]. \end{aligned}$$

In the classical simulation scenario, we assume that the parties have access to shared randomness and a classical communication channel with finite capacity. One subset of this arrangement is where each party uses a finite capacity channel only once. In this case, the procedure can be modeled by a communication-assisted framework as:

7 $$\begin{aligned} \textrm{P}_{\mathrm {\text {LHVC}}}(b|\sigma _\textrm{A},\mathscr {B})= \int d \lambda \, \rho (\lambda )\, \sum _{c_1=0}^{c_1^{\textrm{max}}}\sum _{c_2=0}^{c_2^{\textrm{max}}} \textrm{P}_{C_1}(c_1|\mathscr {B},\lambda )\textrm{P}_{C_2} (c_2|c_1, \sigma _A,\lambda ) \textrm{P}_\textrm{B}(b|c_2,\mathscr {B},\lambda ). \end{aligned}$$

Here $c_1^{\textrm{max}}$ and $c_2^{\textrm{max}}$ are the lengths of the messages, while both are assumed to be finite. $\textrm{P}_{C_1}(c_1|\mathscr {B},\lambda )$ denotes the probability that Bob sends the classical message $C_1 =c_1$ to Alice, conditioned on the measurement $\mathscr {B}$ and shared randomness $\lambda$. Similarly, $\textrm{P}_{C_2} (c_2|c_1, \sigma _A,\lambda )$ represents the probability that Alice sends the classical message $C_2=c_2$ back to Bob, conditioned on her state $\sigma _\textrm{A}$, on the bit $c_1$, and on the value $\lambda$. Finally, $\textrm{P}_\textrm{B}(b|c_2,\mathscr {B},\lambda )$ denotes the probability of Bob obtaining the measurement outcome *b* conditioned on the measurement $\mathscr {B}$, on the value $\lambda$ of the shared random variable, and the bit $c_2$ received from Alice. In our design, $c_1^{\textrm{max}}=1;c_2^{\textrm{max}}=d$. Additionally, we let the protocol be occasionally aborted during some runs. This is indicated by setting $c_2=d$ and the output as $b=$*e*. By excluding these runs, we define:

8 $$\begin{aligned} \tilde{\textrm{P}}_{\mathrm {\text {LHVC}}}(b|\sigma _\textrm{A},\mathscr {B}):= \frac{\textrm{P}_{\mathrm {\text {LHVC}}}(b|\sigma _\textrm{A},\mathscr {B})}{1-\mathrm {P_{LHVC}}(e|\sigma _\textrm{A},\mathscr {B})}. \end{aligned}$$

With this, we state that the model exactly simulates quantum theory if:

9 $$\begin{aligned} \tilde{\textrm{P}}_{\mathrm {\text {LHVC}}}(b|\sigma _\textrm{A},\mathscr {B}) =\textrm{P}_{\textrm{Q}}(b | \sigma _\textrm{A} , \mathscr {B}), \end{aligned}$$

for any arbitrary possible state $\sigma _\textrm{A}$ and measurement $\mathscr {B}$. For the two-dimensional case, such exact models exist, while for $d\ge 3$, the question remains open. Approximate strategies have been proposed in ^13,14^, but systematic studies of their accuracy have not been carried out. Here, we analyze the exact bi-dimensional models^9,10,18,19^ from a novel perspective, identify their key ingredients, and devise a protocol capable of simulating quantum statistics for projective measurements and pure states exactly in $d=2$, and with high accuracy in higher dimensions, $d= 3$ and $d=4$, where accuracy is quantified by the TVD between the protocol statistics and the corresponding quantum predictions.

To this end, we first analyze the $d=2$ PRTQ protocol as presented in^10^ in the case of projective measurements:

- **Protocol Renner–Tavakoli–Quintino (PRTQ)** Setup: Alice and Bob are provided, respectively, with the full description of a state represented by the Bloch vector $\vec {x}$, corresponding to the quantum state $\sigma _\textrm{A}=\frac{I+\vec {x}\cdot \vec {\sigma }}{2}$, and the complete description of a projective measurement characterized by Bloch vectors $\{\vec {y}_b\}$ for the outcomes $b\in \{0,1\}$ ($\vec {y}_0= -\vec {y}_1$) with the vectors normalized as $|\vec {x}|=|\vec {y}_b|=1$. The measurement operators associated with Bob’s measurement are expressed as $B_b = \frac{I+\vec {y}_b\cdot \vec {\sigma }}{2}$. Additionally, both parties have access to two independent sources of randomness, generating real three-dimensional unit vectors $\vec {\lambda }_0$ and $\vec {\lambda }_1$, sampled uniformly from the unit sphere $\mathbb {S}_2$.

Protocol:

1. Bob,
  - Selects one of his measurement vectors $\vec {y}_b$ (for a projective measurement, the choice is arbitrary)
  - He sets $c_1 = H(|\vec {y}_b\cdot \vec {\lambda }_0| - |\vec {y}_b\cdot \vec {\lambda }_1|)$, where *H*(*x*) is Heaviside step function defined as: $H(x)=1$ if $x\ge 0$ and $H(x)=0$ otherwise;
  - He sends the classical message $c_1$ to Alice.
2. Alice,
  - receives the message $c_1$, and computes $c_2=H(\vec {x}\cdot \vec {\lambda }_{c_1})$.
  - She sends the classical bit $c_2$ to Bob
3. Bob,
  - receives the message $c_2$, and implements the transformation $\vec {\lambda }_{c_1} \mapsto (-1)^{1+c_2}\vec {\lambda }_{c_1} =\vec {\lambda }^\prime$. In other words, he flips the sign of vector $\vec {\lambda }_{c_1}$ if $c_2=0$;
  - Finally, Bob reports the output *b*, with probability
    10 $$\begin{aligned} \textrm{P}(b|\vec {\lambda }^\prime ,\vec {y}_b)= \frac{ H(\vec {y}_b\cdot \vec {\lambda }^\prime )}{ H(\vec {y}_b\cdot \vec {\lambda }^\prime ) - H(-\vec {y}_b\cdot \vec {\lambda }^\prime )}. \end{aligned}$$

In step 1, $c_1$ can be regarded as a choice of the shared vector, $\vec {\lambda }_0$ or $\vec {\lambda }_1$, that is more aligned with the direction of $\vec {y}_b$, hence the name *choice method*.

However, an essential implication of this step is that it effectively results in sharing a random vector $\vec {\lambda }$ sampled according to the distribution

11 $$\begin{aligned} \rho (\vec {\lambda }|\vec {y}_b)=\mathscr {N} |\vec {\lambda } \cdot \vec {y} _b|. \end{aligned}$$

Here, $\mathscr {N}$ is a normalization factor^18^. This construction is based on the principles of the rejection method, a standard statistical technique for generating random variables from a target distribution. A detailed explanation is provided in SM, where we demonstrate that it is possible to implement a protocol based on the rejection method in place of the original choice method. The only modification is that Alice and Bob now share a single instance of $\vec {\lambda }$, and the message $c_{1}$ instead of selecting one random vector, informs Alice whether the current iteration should be accepted or rejected. All other steps of the protocol remain unchanged. It can be proved that the two versions of the protocol are equivalent in terms of both the distribution of outcomes and the average number of shared samples^18^. Therefore, from now on, we focus the analysis on the shared distribution $\rho (\vec {\lambda }|\vec {y}_b)$ and exploit the rejection method across different protocols to sample from the corresponding target distributions.

The quantity $|\vec {\lambda } \cdot \vec {y} _b|$, defined as the absolute value of the cosine similarity, serves as a measure of similarity between measurements $\Lambda = \{\Lambda _{\vec {\lambda }},\Lambda _{-\vec {\lambda }}\}$ and $\mathscr {B}=\{B_{\vec {y}_b},B_{-\vec {y}_b}\}$, expressed as:

12 $$\begin{aligned} |\vec {\lambda }\cdot \vec {y}_b| = |\textrm{P}(\Lambda _{ \vec {\lambda }} | B_{ \vec {y}_b}) - \textrm{P}(\Lambda _{ - \vec { \lambda }} | B_{ \vec {y}_b})|, \end{aligned}$$

where each projector is labeled by its Bloch vector. Here, $\textrm{P}(\Lambda _{ \vec {\lambda }} | B_{ \vec {y}_b})$ denotes the probability that measuring the state represented by $B_{ \vec {y}_b}$ in the basis $\Lambda$, yields the outcome $\vec {\lambda }$ associated with the projector $\Lambda _{ \vec {\lambda }}$. The magnitude of this probability difference quantifies the measurement correlation: a value of 1 indicates perfect alignment between $\Lambda$ and $\mathscr {B}$, while a value of 0 signifies complete orthogonality, i.e., that the measurement outcome from one basis provides no information about the other basis. Thus, PRTQ, along with the ones introduced^9,18,19^, uses randomly selected projective measurements $\Lambda$ as a shared random variable, with sampling weights determined by their similarity to the target measurement $\mathscr {B}$. For dimension $d=2$, this similarity is uniquely defined by the scalar quantity $|\vec {\lambda }\cdot \vec {y}_b|$.

To extend this concept to higher dimensions, consider two random variables $O_\Lambda ,O_ \mathscr {B}\in \{0,\dots , d-1\}$ representing the outcome indices of the projective measurements given by $\Lambda =\{\Lambda _i\}_{i=0}^{d-1}$ and $\mathscr {B}=\{B_j\}_{j=0}^{d-1}$, respectively. Let $\textrm{P}(O_\Lambda = i|O_\mathscr {B}=j)$ denote the conditional probability of obtaining outcome *i* under the measurement $O_\Lambda$ given that the outcome *j* was observed under the measurement $\mathscr {B}$. A natural generalization of the similarity measure in Eq. (12) is given by:

13 $$\begin{aligned} D_{\Lambda ,\mathscr {B}}(i,j):=\textrm{P}(O_\Lambda = i|O_\mathscr {B}=j) - \textrm{P}(O_\Lambda \ne i | O_\mathscr {B} = j) =2 \textrm{P}(O_\Lambda = i|O_\mathscr {B}=j) -1. \end{aligned}$$

Thus, the condition that $D_{\Lambda ,\mathscr {B}}(i,j) \ge 0$ is equivalent to

14 $$\begin{aligned} \textrm{P}(O_\Lambda = i|O_\mathscr {B}=j)\ge \frac{1}{2}. \end{aligned}$$

For each outcome *j*(*i*), there is at most one outcome *i*(*j*) that satisfies this condition for any possible $O_\Lambda$ and $O_\mathscr {B}$. (Observe that only for two-outcome random variables $O_\Lambda$ and $O_\mathscr {B}$ such an outcome always exists.)

A large $D_{\Lambda ,\mathscr {B}}(i,j)$ indicates that whenever the outcome *j* is observed under $O_\mathscr {B}$, the outcome *i* under $O_\Lambda$ occurs with high probability. In the limiting cases, where for each *j*, there exist an outcome *i* such that $D_{\Lambda ,\mathscr {B}}(i,j)=1$, the statistics of $O_\Lambda$ and $O_\mathscr {B}$ become effectively equivalent, *i.e.*, knowing the outcome $j\in O_\mathscr {B}$ determines $i\in O_\Lambda$ with absolute certainty. On the other hand, if $D_{\Lambda ,\mathscr {B}}(i,j) < 0$ for at least one outcome $j\in O_\mathscr {B}$, this implies that conditioning on $O_\mathscr {B}=j$ does not make any single outcome $i\in O_\Lambda$ more likely than the combined probabilities of all the other outcomes. In such a case, the outcomes of $O_\mathscr {B}$ fail to provide full information about the statistics of $O_\Lambda$.

Before presenting the explicit steps of the protocol, we outline its key components. First, the conditional probabilities are defined, analogously to the two-dimensional case, as $\textrm{P}(O_\Lambda = i|O_\mathscr {B}=j) = \text {Tr}[\Lambda _i B_j]$. Second, we restrict our attention only to informative events, *i.e.*, those in which the randomly selected shared basis $\Lambda$ satisfies the condition that, for each possible outcome *j* of $O_{\mathscr {B}}$, there exists at least one outcome *i* of $O_{{\Lambda }}$ such that $D_{\Lambda ,\mathscr {B}}(i,j)\ge 0$. If this condition is not met, the event is rejected (in the case $d=2$, all events satisfy this condition.).

Third, we must specify a shared probability distribution between Alice and Bob for the accepted events. This probability should depend on the similarity function $D_{\Lambda ,\mathscr {B}}(i,j)$ for potentially *d* different possible pairs (*i*, *j*) and it should reduce to a form proportional to $|\vec {\lambda }\cdot \vec {y}_b|$ for $d=2$ in accordance with Eq. (12). A simple functional that satisfies this requirement is $D_{\Lambda ,\mathscr {B}}(i^*,j^*)^{\alpha _d}$, where $(i^*,j^*):=\text {argmin}_{(i,j)} D_{\Lambda ,\mathscr {B}}(i,j)$, and $\alpha _d$ is a parameter that depends on the dimension *d*. For $d=2$, $\alpha _2=1$. For higher dimensions, we observe that from all pairs (*i*, *j*), the leading dependence by far is on the term $D_{\Lambda ,\mathscr {B}}(i^*,j^*)$, and we find empirically that the optimal exponents in $D_{\Lambda ,\mathscr {B}}(i^*,j^*)^{\alpha _d}$ are $\alpha _3=1/2$ and $\alpha _4=1/4$, suggesting a general scaling of $\alpha _d=2^{2-d}$. Note that as the dimension *d* increases, the shared distribution becomes increasingly uniform, indicating that the accepted events tend to be more equally informative. While more sophisticated variants involving richer dependencies on $D_{\Lambda ,\mathscr {B}}(i,j)$ values are conceivable and worth exploring, our investigation of various alternative functionals, incorporating contributions of different (*i*, *j*) pairs, has shown no improvement in TVD. We obtain that the simple power-law form already captures the key quantum features and yields a protocol that remains robust across different scenarios.

Finally, we have analyzed the role of the threshold parameter 1/2 in the rejection condition $H(\text {Tr}[\Lambda _{i^*}B_{j^*}]-\frac{1}{2})$. To this end, We consider a distribution $\rho _d(\Lambda |\mathscr {B})$ proportional to $(\text {Tr}[\Lambda _{i^*}B_{j^*}]-\Delta )^{\alpha _d}H(\text {Tr}[\Lambda _{i^*}B_{j^*}]-\Delta )$ with a tunable parameter $\Delta$ close to 1/2. The numerical computation of the TVD reveals that the accuracy reaches its maximum when $\Delta =1/2$, in agreement with our theoretical expectation. The numerical results are provided in Table 2 of SM.

Therefore, combining the elements discussed above, the shared distribution can be written as

15 $$\begin{aligned} \rho _d(\Lambda |\mathscr {B})=\, \mathscr {N}_d H(\text {Tr}[\Lambda _{i^*}B_{j^*}]-1/2)\times \left( \text {Tr}[\Lambda _{i^*}B_{j^*}]-\frac{1}{2}\right) ^{\alpha _d}, \end{aligned}$$

where $\mathscr {N}_d$ is the normalization constant that depends on *d*.

We are now ready to present the explicit steps of our protocol.

- **P1** Setup: Alice receives the full description of a pure state $\sigma _\textrm{A} \in \mathscr {L}(\mathscr {H}_\textrm{A})$ and Bob receives the full description of a projective measurement $\mathscr {B}=\{B_i\}_{i=0}^{d-1}$ and both parties have access to a source that generates the description of a random projective measurement expressed by $\Lambda =\{\Lambda _j\}_{j=0}^{d-1}$. In addition, Bob has access to a random number generator that samples $u\in [0,1]$ uniformly, and sets an appropriate scaling factor $M_d$, required for applying the rejection sampling method (See SM.).

Protocol:

1. Bob,
  - Calculates $$\text {Tr}[\Lambda _{i^*}B_{j^*}]$$.
  - He sets $$c_1=H( \frac{(\text {Tr}[\Lambda _{i^*}B_{j^*}]-\frac{1}{2})^{\alpha _d}\times H(\text {Tr}[\Lambda _{i^*}B_{j^*}]-\frac{1}{2})}{M_d}-u)$$.
  - He sends the bit $c_1$ to Alice.
2. Alice,
  - receives the bit $c_1$. If $c_1=0$, she sets $c_2=d$. Otherwise, she sets $c_2=\arg {\max _{i}\{\text {Tr} [\Lambda _i\sigma _\textrm{A}]\}}$.
  - She sends $c_2$ to Bob.
3. Bob reports $b = \arg {\max _j \{\text {Tr}{[B_j\Lambda _{c_2} ]}\}}$ if $c_2\ne d$. Otherwise, he reports interruption of the protocol by setting $b=e$.

The operational effect of the protocol can be modeled by:

16 $$\begin{aligned} \tilde{\textrm{P}}_{\text {LHVC}}(b|\sigma _\textrm{A},\mathscr {B})= \int _{SU(d)} d \Lambda \rho _d(\Lambda | \mathscr {B}) \sum _{c_2=0}^{d-1} \textrm{P}_{C_2}(c_2|\Lambda , \sigma _\textrm{A}) \textrm{P}(b|c_2,\mathscr {B} , \Lambda ). \end{aligned}$$

The initial message $c_1$ serves to share the probability density $\rho _d(\Lambda | \mathscr {B})$ with Alice. The instructions for the following steps are then represented by $\textrm{P}_{C_2}(c_2|\Lambda , \sigma _\textrm{A})$ and $\textrm{P}(b|c_2,\mathscr {B}, \Lambda )$ within the integral.

For $d=2$, one can see that P1 is equivalent to PRTQ with the only difference that the rejection sampling method is used instead of the choice method (see SM.). This becomes apparent by observing that: (*i*) choosing a random vector $\vec {\lambda }$ with $|\vec {\lambda }|=1$ is equivalent to choosing a random projective measurement $\Lambda$. (*ii*) Step 2 reduces to simply comparing $u \le |\cos {\theta }|$ which gives the distribution $\rho (\vec {\lambda }\big | \vec {y}_b)$ proportional to $|\vec {\lambda }.\vec {y}_b|=|\cos {\theta }|$ where $D_{\Lambda ,\mathscr {B}}( i^*,j^*)=|\cos {\theta }|$. (*iii*) The condition $D_{\Lambda ,\mathscr {B}}( i^*,j^*)\ge \frac{1}{2}$ is always satisfied. (*iv*) In the following steps, sending the message $\arg {\max _{i}\{\text {Tr} [{\Lambda _i\sigma _\textrm{A}}}]\}$ becomes equivalent to Bob inverting the shared vector as $\vec {\lambda } \mapsto (-1)^{1+c_2}\vec {\lambda }:=\vec {\lambda }^\prime$ depending on Alice’s message, and $\arg {\max _j\{\text {Tr}{[B_j\Lambda _{c_2} ]}\}}$ becomes equivalent to selecting the outcome *b* such that $H(\vec {\lambda } \cdot \vec {y}_b)=1$, which corresponds to Step 4 of PRTQ.

### Entanglement scenario

The entanglement scenario for projective measurements can be expressed as follows: Two parties, each having access to a subsystem of a maximally entangled state $|\Omega \rangle :=\frac{1}{\sqrt{d}} \sum _i^d | ii \rangle$. They perform a local projective measurement on their subsystems, denoted by $\mathscr {A}$ for Alice and $\mathscr {B}$ for Bob. The joint outcome statistics are governed by the Born rule: $\textrm{P}_{\textrm{Q}}(a,b| \mathscr {A}, \mathscr {B}, |\Omega \rangle ) = \text {Tr}[A_a \otimes B_b | \Omega \rangle \langle \Omega |]$, where *a* and *b* are the outcomes and $A_a\in \mathscr {L}(\mathscr {H}_\textrm{A})$ and $B_a\in \mathscr {L}(\mathscr {H}_\textrm{B})$ are the corresponding projectors in $\mathscr {A}$ and $\mathscr {B}$, respectively.

Here, a communication-assisted model can be represented by:

17 $$\begin{aligned} \textrm{P}_{\text {LHVC}}(a,b|\mathscr {A},\mathscr {B})= \int \rho (\lambda ) \sum _{c_1=0}^{c_1^{\textrm{max}}}\sum _{c_2=0}^{c_2^{\textrm{max}}} \textrm{P}_{C_1}(c_1|\mathscr {B},\lambda )\textrm{P}_{\textrm{A},C_2}(a,c_2|c_1, \mathscr {A},\lambda ) \textrm{P}_\textrm{B}(b|c_2,\mathscr {B},\lambda ). \end{aligned}$$

With a slight modification from the model provided in Eq. (7). In the entanglement scenario, Alice reports an outcome *a* and sends the classical bit $c_2$ to Bob, with probability $\textrm{P}_{\textrm{A},C_2}(a,c_2|c_1, \mathscr {A},\lambda )$. The objective is to construct a model with finite communication such that:

18 $$\begin{aligned} \tilde{\textrm{P}}_{\text {LHVC}}(a,b|\mathscr {A},\mathscr {B}):=\frac{\textrm{P}_{\text {LHVC}}(a,b|\mathscr {A},\mathscr {B})}{1-\textrm{P}_{\text {LHVC}}(e,e|\mathscr {A},\mathscr {B})} =\textrm{P}_{\textrm{Q}}(a,b| \mathscr {A}, \mathscr {B}, |\Omega \rangle ), \end{aligned}$$

for any arbitrary projective measurements $\mathscr {A}$ and $\mathscr {B}$.

This scenario is closely connected to the PM scenario, discussed earlier via the Choi–Jamiołkowski isomorphism^15,16^, which establishes a correspondence between positive trace-preserving maps $\mathscr {E}: \mathscr {L}(\mathscr {H}_\textrm{A}) \rightarrow \mathscr {L}(\mathscr {H}_{\textrm{B}})$ and a quantum state. Specifically, the Choi matrix associated to $\mathscr {E}$ is defined as $J(\mathscr {E}):=\sum _{i,j=0}^{d-1} \mathscr {E}(|i\rangle \langle j |) \otimes |i\rangle \langle j | \in \mathscr {L}(\mathscr {H}_\textrm{A} \otimes \mathscr {H}_{\textrm{B}})$ and captures the full action of $\mathscr {E}$, allowing one to translate results between the PM and entanglement scenarios. As a corollary, by applying this isomorphism to the identity map $\mathscr {I}$ acting on $A_a\in \mathscr {L}(\mathscr {H}_\textrm{A})$, and taking the partial trace over the subspace $\textrm{B}$, one obtains:

19 $$\begin{aligned} \mathscr {I}(A_a)=A_a = \text {Tr}_\textrm{B}[\mathscr {I} \otimes A_a^T J(\mathscr {I})], \end{aligned}$$

where $J(\mathscr {I}):=\sum _{i,j=0}^{d-1} |i\rangle \langle j | \otimes |i\rangle \langle j | \in \mathscr {L}(\mathscr {H}_\textrm{A} \otimes \mathscr {B}_B)$ is the Choi matrix of the identity map $\mathscr {I}\in \mathscr {L}(\mathscr {H}_\textrm{A})$, and $A_a^T :=\sum _{i,j=0}^{d-1} \langle j |A_a|i\rangle |i\rangle \langle j | \in \mathscr {L}(\mathscr {H}_\textrm{B})$ is the transpose of $A_a$ in the computational basis. This result implies that any protocol simulating the quantum PM can be converted to one simulating the entanglement scenario, and vice versa. To establish the link, first notice that:

20 $$\begin{aligned} \textrm{P}_{\textrm{Q}}(a,b| \mathscr {A}, \mathscr {B}, |\Omega \rangle )= \textrm{P}_{\textrm{Q}}(b|a,\mathscr {A}, \mathscr {B} ,|\Omega \rangle )\textrm{P}_{\textrm{Q}}(a|\mathscr {A} , \mathscr {B}, |\Omega \rangle ) =\frac{1}{d}\textrm{P}_{\textrm{Q}}(b|a,\mathscr {A}, \mathscr {B} ,|\Omega \rangle ). \end{aligned}$$

The second equality follows because the probability of each measurement outcome on subsystem A is independent of subsystem B and is equal to $\frac{1}{d}$. Now, by using the Choi–Jamiołkowski isomorphism:

21 $$\begin{aligned} \textrm{P}_{\textrm{Q}}(b|a,\mathscr {A}, \mathscr {B} ,|\Omega \rangle )= \text {Tr}[B_b\text {Tr}_\textrm{A}[\mathscr {I}\otimes A_a J(\mathscr {I})] ] =\text {Tr}[B_bA_a^T] = \textrm{P}_{\textrm{Q}}(b|A_a^T ,\mathscr {B}). \end{aligned}$$

Therefore, if one considers a projective measurement $\mathscr {A}^T:= \{A^T_{a}|a\in \{0,1,\dots ,d-1\}\}$, where $A^T_{a=0}=\sigma _\textrm{A}^T$ and the projectors corresponding to the outcomes $a\ne 0$ are arbitrary, the problem of simulating the entanglement scenario becomes linked to the simulation of the PM scenario by conditioning the outcomes on $a=0$ as follows:

22 $$\begin{aligned} \text {P}_{\textrm{Q}}&(a=0,b| \mathscr {A}^T, \mathscr {B}, |\Omega \rangle )=\text {P}_{\textrm{Q}}(a=0,b| \mathscr {A}, \mathscr {B}^T, |\Omega \rangle )=\frac{1}{d}\textrm{P}_{\textrm{Q}}(b|\sigma _\textrm{A} ,\mathscr {B}), \end{aligned}$$

where we have used that $\text {Tr}[B_b A^T_a]~=~\text {Tr}[B^T_b A_a]$, *i.e,* that one can equivalently consider the measurement $\mathscr {A}$ with $A_{a=0} =\sigma _{\text {A}}$, and instead, transpose the elements of $\mathscr {B}$. Now, starting from a protocol originally simulating the entanglement scenario, setting its input to $(\mathscr {A}^T,\mathscr {B})$ or $(\mathscr {A},\mathscr {B}^T)$ and accepting its outputs conditioned on $a=0$, will produce the statistics of the PM scenario for $(\sigma _\text {A},\mathscr {B})$. Inversely, if a protocol is originally designed for the PM scenario, the parties run it for the inputs $(A_a^T,\mathscr {B})$ or $(A_a,\mathscr {B}^T)$, where *a* is selected at each run by rolling an unbiased die of *d* outcomes. This leads to the simulation of the entanglement scenario for $(\mathscr {A},\mathscr {B})$^14^. With this in hand, we explicitly propose the protocol for simulating the entanglement scenario as follows:

- **P1(entanglement mode)** Setup: Alice and Bob are respectively given the full description of two projective measurements $\mathscr {A}=\{A_i\}_{i=0}^{d-1}$ and $\mathscr {B}=\{B_i\}_{i=0}^{d-1}$; and both also have access to a source of random orthonormal basis generator, producing projective measurements expressed by $\Lambda =\{\Lambda _j\}_{j=0}^{d-1}$. Additionally, Bob has access to a random number generator that samples $u\in [0,1]$ uniformly, and sets an appropriate scaling factor $M_d$ (See SM.).

Protocol:

1. Bob,
  - calculates $$\text {Tr}[\Lambda _{i^*}B_{j^*}^T]$$.
  - He sets $$c_1=H( \frac{(\text {Tr}[\Lambda _{i^*}B_{j^*}^{T}]-\frac{1}{2})^{\alpha _d}\times H(\text {Tr}[\Lambda _{i^*}B_{j^*}^{T}]-\frac{1}{2})}{M_d}-u)$$.
  - He sends the bit $c_1$ to Alice.
2. Alice,
  - receives the bit $c_1$. If $c_1=0$, she sets $c_2=d$ and reports the interruption of the protocol by setting $a=e$. Otherwise, she rolls an unbiased die of *d* possible outcomes $\{0,\dots ,d-1\}$ and reports the output of the die as *a*. Then, she sets $c_2=\arg {\max _{i}\{\text {Tr}[{\Lambda _iA_a}}]\}$.
  - She sends $c_2$ to Bob.
3. Bob reports $b = \arg {\max _j\{\text {Tr}{[B^T_j\Lambda _{c_2} ]}\}}$ if $c_2\ne d$. Otherwise, he reports the interruption of the protocol by setting $b=e$.

## Results

To compare the performance of the protocols, we conducted a randomized numerical study by running each protocol over a set of *n* randomly selected pairs of inputs: $\{(\sigma _{A},\mathscr {B})_i\}_{i=1}^n$ for the PM scenario and $\{(\mathscr {A},\mathscr {B})_i\}_{i=1}^n$ for the entanglement scenario. For each setup, the size of the samples of $\Lambda$ is denoted by $N_{\textrm{ini}}$ and the number of the outputs by $N_{\textrm{out}}$. For this purpose, we have used the method provided by^23^ to generate uniformly distributed $\Lambda$, $\sigma _\textrm{A}$, $\mathscr {A}$, and $\mathscr {B}$. To assess the performance of the protocols, we use TVD to quantify the distance between the probability distribution predicted by quantum theory and the probability distributions obtained via the classical protocols, which reads

23 $$\begin{aligned} \delta =\frac{1}{2n}\sum _{i=1}^n\sum ^d_{b=1} |\tilde{\textrm{P}}_{\text {LHVC}}(b | \sigma ^{i}_\textrm{A}, \mathscr {B}^{i})-\textrm{P}_{\textrm{Q}}(b | \sigma ^{i}_\textrm{A} , \mathscr {B}^{i})|, \end{aligned}$$

for the PM scenario, and

24 $$\begin{aligned} \delta =\frac{1}{2n}\sum _{i=1}^n\sum ^d_{a,b=1} |\tilde{\textrm{P}}_{\text {LHVC}}(a,b |\mathscr {A}^{i} , \mathscr {B}^{i})-\textrm{P}_{\textrm{Q}}(a,b | \mathscr {A}^{i}, \mathscr {B}^{i})| \end{aligned}$$

for the entanglement scenario.

Although the KLD is also a possible measure of discrepancy between probability distributions, it can become unbounded when one of the probabilities approaches zero while the other remains nonzero, as occurs, for example, in the $\phi -$parameterized setup analyzed below. As a result, such instances can contribute disproportionately to the average and thereby skew the evaluation of the typical performance of the protocols. For this reason, TVD provides a more stable and suitable figure of merit in our setting.

Additionally, although the PM and entanglement scenarios are related through the Choi–Jamiołkowski isomorphism, they are defined on fundamentally different sample spaces. Accordingly, each protocol is studied separately in the two scenarios, since performance in one does not necessarily imply comparable performance in the other.

The numerical uncertainty in our estimation of $\delta$ primarily stems from two sources: the finite number of input setups *n*, and the finite sample of output events, $N_{\text {out}}$. Furthermore, due to computational limitations, a common set of shared randomness samples was used for all inputs within each protocol. This introduces complex correlations between the TVDs from different input setups, making a rigorous error estimation challenging. With these considerations in mind, we characterize the deviations due to numerical sampling only by the empirical estimate $std_n/\sqrt{n}$ across randomized inputs.

To carry out the runs, we use the rejection method in protocols P1, PRUD-1, and PRUD-2. For P1, we set $M_2=0.5$, and $M_3=M_4=0.7$, and for PRUD-1 and PRUD-2, $M_d=1$ for all *d*’s. The details of how to choose this parameter are provided in SM.

### d=2

We have run the protocols for $n=100$ uniformly distributed random input setups, with $N_{\textrm{out}}\approx 2.5 \times 10^5$ outputs for both the PM and the entanglement scenarios. The results reveal that, as expected, P1, PMON-1, PMON-2A, and PRUD-1 simulate quantum predictions exactly, with a small deviation of order $\delta \approx 0.001$ that is due to the limited sample size. The exactness of the protocols is further supported by examining the scaling of the $\delta$ with the number of samples, which decreases proportionally to $1/\sqrt{N_{\textrm{out}}}$, as it should, for any arbitrarily chosen input.

We also confirm that PMON-2B and PRUD-2 are not exact, which is reflected in the values of the $\delta$. Full results are reported in Table 1 of SM.

### d=3

**Fig. 1:**
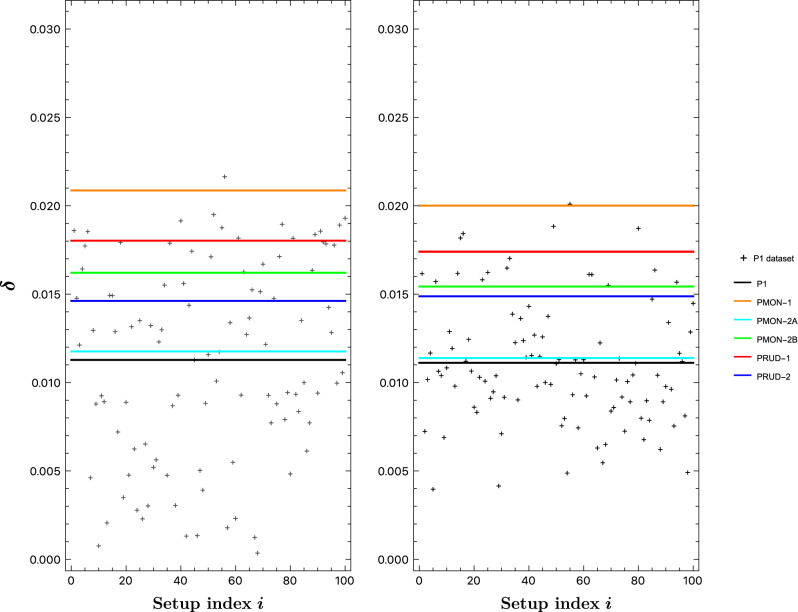
Average performance of the protocols for $d=3$ is shown as lines overlaid on the instances produced by running P1, for $n=100$ randomized inputs–on the left for the PM scenario and on the right for the entanglement scenario. The horizontal axis is the setup index $i\in \{1,\cdots ,n\}$ of the input setup $(\sigma _\textrm{A},\mathscr {B})_i$ for the PM, and $(\mathscr {A},\mathscr {B})_i$ for the entanglement scenario.

We have analyzed $n=100$ uniformly distributed random input setups, with $N_{\textrm{out}}\approx 1.2 \times 10^5$ outputs for both the PM and the entanglement scenarios. Our results show that P1 provides the most accurate predictions among all protocols in both scenarios with the lowest $\delta = 0.011$, with PMON-2A following it closely. Figure 1 summarizes the average performance of all protocols over the randomized inputs studied in this dimension. Complete numerical data are provided in Table 3 of SM.

We also analyze the robustness of the protocols by studying their performance away from random setups and consider structured sources of states and measurements. For the PM scenario, we consider a case, denoted by $\phi -$parameterized setup, where one of the measurement states of $\mathscr {B}$, for example $|b=3\rangle$, has no overlap with the state $|\psi \rangle$ i.e., $|\psi \rangle \in \text {span}\{|b=1\rangle , |b=2\rangle \}$ where $\sigma _\textrm{A} =|\psi \rangle \langle \psi |$. As suggested in^13,14^, this structure parameterized solely by $\phi$, makes it convenient to visualize the performance of the protocols as a function of $\phi$:

25 $$\begin{aligned} |b = 1\rangle&= \cos {\phi }|\psi \rangle + \sin {\phi } |\psi _{\perp }^{(2)}\rangle \nonumber \\ |b = 2 \rangle&= \sin {\phi }|\psi \rangle - \cos {\phi } |\psi _{\perp }^{(2)}\rangle \nonumber \\ |b = 3\rangle&= |\psi _{\perp }^{(3)} \rangle . \end{aligned}$$

**Fig. 2:**
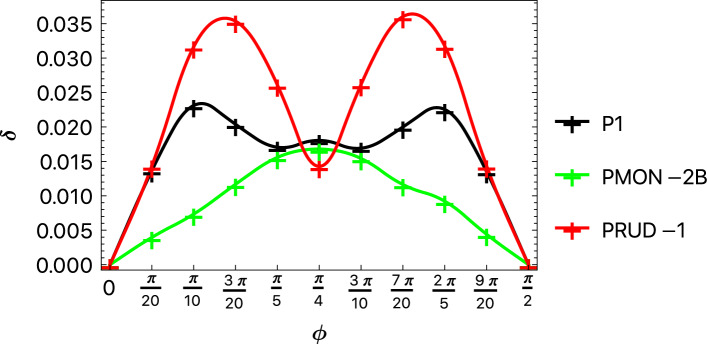
Performance of PMON-2B, PRUD-1, and P1, for the $\phi -$parameterized setup for $d=3$.

**Fig. 3:**
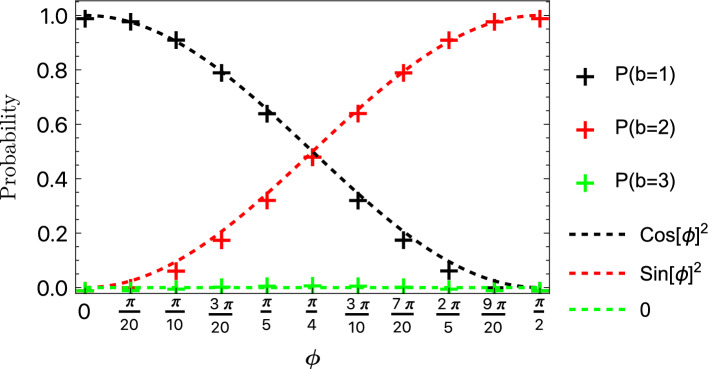
Probability of outcomes of P1 for the $\phi -$parameterized setup for $d=3$, as a function of $\phi$, shown by cross, is compared to the exact values from quantum theory, presented by dashed lines, with $\textrm{P}_{\textrm{Q}}(b = 1\big |\sigma _\textrm{A})=\cos ^2{\phi }$, $\textrm{P}_{\textrm{Q}}(b = 2\big |\,\sigma _\textrm{A})=\sin ^2{\phi }$, and $\textrm{P}_{\textrm{Q}}(b = 3\big |\sigma _\textrm{A})=0$.

Here $|\psi _{\perp }^{(2)}\rangle$ and $|\psi _{\perp }^{(3)}\rangle$ are arbitrary perpendicular states, chosen such that $\{\sigma _\textrm{A},|\psi _{\perp }^{(2)}\rangle \langle \psi _{\perp }^{(2)} |, |\psi _{\perp }^{(3)}\rangle \langle \psi _{\perp }^{(3)} | \}$ forms a valid projective measurement. We have chosen a set of random $\sigma _\textrm{A}$’s and then constructed the measurement accordingly. We have tested the performance of the protocols for 11 different values of $\phi$ ($n=11$) in the range $[0,\pi /2]$. We obtain that PMON-2B and PRUD-2 yield the best values, followed by P1. In Fig. 2, we have plotted the performance of the best, worst, and our protocol. Additionally, in Fig. 3, we plot the results of P1 compared to the exact quantum predictions. As can be seen, P1 approximates the exact values for the three possible outcomes very well.

As a structured entanglement scenario, we consider the configuration provided in^24^. Alice and Bob each receive two different measurements ($n=4$). The measurements for each party consist of a combination of phase shifts and discrete Fourier transforms, followed by measuring in the computational basis. This setup leads to a maximal violation of the local reality constraint by quantum theory as given by the CGLMP inequalities ^24^ (a generalization of the CHSH inequalities for $d\ge 3$). We refer to it as the CGLMP setup. In this case, the best predictions of the quantum probability distributions come from PRUD-2 and PMON-2B in contrast to the randomized case. PMON-1, and P1 follow closely with comparable performance. While the accuracy of P1 remains comparable to the randomized case, the accuracy of PMON-2A, a top performer in that setup, drops significantly here. The increase in its error $\delta$ from the randomized case is approximately double the increase observed for P1, which casts it as the second-worst protocol for this setup. As an additional performance benchmark, we calculated the CGLMP inequality value for each protocol, which can be compared against the quantum value 2.87. These two structured setups show significant reordering of the best-performing protocols, while P1 retains comparatively robust performance. Notably, in both the $\phi -$parameterized case and the CGLMP setup, the protocols that perform best on randomized inputs exhibit a noticeable deterioration in their ability to reproduce the quantum statistics. More specifically, the maximum TVD values for P1, PMON-2A, and PRUD-1 are numerically observed in these structured configurations (consistently with the empirical observations reported in ^13^). Furthermore, we found that PRUD-1 exhibits its poorest performance in CGLMP setup. The full numerical results for these two special setups are reported in Tables 4 and 5 of the SM.

### d=4

The performance of all protocols is tested for $n=100$ and $N_{\textrm{out}} \approx 1 \times 10^5$ randomized inputs, for both scenarios.

**Fig. 4:**
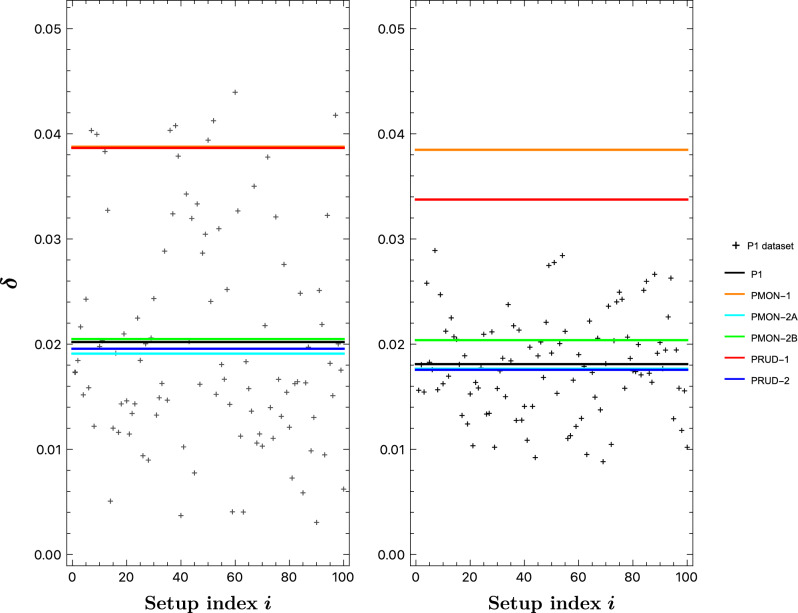
Average performance of the protocols for $d=4$ is shown as lines overlaid on the instances produced by running P1, for $n=100$ randomized inputs—on the left for the PM scenario and on the right for the entanglement scenario. The horizontal axis is the setup index $i\in \{1,\ldots ,n\}$ of the input setup $(\sigma _\textrm{A},\mathscr {B})_i$ for the PM, and $(\mathscr {A},\mathscr {B})_i$ for the entanglement scenario.

Figure 4 shows that PMON-2A, PRUD-2, and P1 are the top-performing protocols, with a difference in their performance, comparable to $std_n/\sqrt{n}$. From $d=3$ to $d=4$, while the accuracy of PMON-1 and PRUD-1 degrades significantly, the performances of the other protocols become numerically closer to each other (compare Tables 3 and 6 of SM.).

**Fig. 5:**
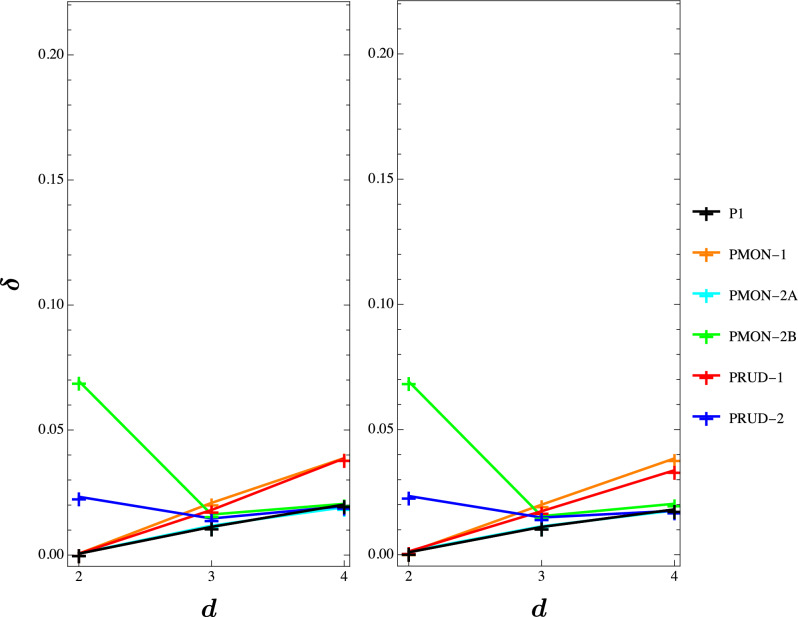
Average performance of the protocols as a function of *d*—on the left for the PM, and on the right for the entanglement scenario.

In Fig. 5, the average performance of the protocols for the randomized input setups is plotted as a function of dimension *d*. It is important to mention $N_{\textrm{out}}/N_{\textrm{ini}}$ gets smaller with increasing *d*. As it is shown, protocols P1 and PMON-2A achieve the best performance, on average, while P1, also, turns out to be robust against changing the inputs structure.

These protocols can, in principle, be extended to arbitrary dimensions. However, increasing the dimensionality poses significant challenges in terms of computational resources. Notably, the decrease of the ratio $N_{\textrm{out}} / N_{\textrm{ini}}$ with higher *d*, makes the statistical analysis considerably more demanding. While we expect the trends observed to persist for higher dimensions, a comprehensive numerical investigation remains for future research.

## Discussion and conclusions

The exact protocols for qubits introduced in^9,10,17,18,20^ rely heavily on the unique geometric structure of the qubit space, which poses significant challenges when attempting to generalize them to higher dimensions. In this work, we have identified the essential ingredients underlying these protocols and reformulated them in a geometry-independent framework, paving the way for generalization beyond $d=2$.

Our central idea has been to regard the classical simulation process as a weighted sampling over shared randomness. Building on this, we have developed a novel geometry-independent protocol that defines a distribution over *SU*(*d*) and provides a new classical communication strategy that closely approximates quantum probability distributions.

To evaluate its performance, we conducted a randomized numerical study comparing our protocol with five existing ones for $d=2,3,4$. Our results show the protocol is exact for $d=2$, and performs among the best for $d=3$ and $d=4$. In particular, it attains the lowest mean TVD for $d=3$ in both the PM and entanglement scenarios. For both $d=3$ and $d=4$, its performance remains statistically comparable to that of the other leading protocols, with differences of the order of $std_n/\sqrt{n}$. Moreover, its ability to maintain comparatively low TVD across both randomized and structured input configurations indicates that it is the most robust protocol considered in this study.

Our analysis also shows that the protocols behave qualitatively differently in two specific structured configurations: the $\phi -$parameterized setup and the CGLMP setup. These cases induce a significant reordering in the ranking of the best-performing protocols relative to the randomized instances. Notably, the $\phi -$parameterized setup yields the largest numerical deviation from the quantum predictions for P1, PMON-2A, and PRUD-1, and in the entanglement scenario, the CGLMP setup yields the worst-case performance for PRUD-1. Since any exact simulation protocol must reproduce the quantum statistics for all valid inputs, these observations suggest that such structured cases are possibly especially relevant for future analytical work and may provide useful insight into the ingredients required for an exact higher-dimensional protocol.

The similarity function $D_{\Lambda ,\mathscr {B}}(i,j)$ introduced in Eq. (13) contains several independent degrees of freedom for $d\ge 3$, unlike the qubit case. To account for this, we explored alternative probability distributions incorporating different contributions of index pairs (*i*, *j*). However, within the range of cases studied here, we have not observed any improvement in TVD averaged over randomized inputs. Nevertheless, we expect that additional terms may become relevant as *d* increases. This suggests that the simple form considered in this work may already be the dominant element relevant to the simulation, although additional contributions may start to play a role in higher dimensions.

At the same time, we emphasize that these conclusions are based on finite-sample numerical evidence and do not yet provide analytical performance guarantees for $d\ge 3$. Deriving such guarantees remains an important open problem. Because the complexity of the relevant structure grows rapidly with the dimension, a purely analytical treatment becomes increasingly difficult. For this reason, machine-learning-based approaches may provide a useful complementary tool. Recent work has already shown that such methods can help identify classical simulability of quantum distributions^25^ and construct communication-assisted hidden-variable models^26^. In this direction, our preliminary results suggest that suitably trained classical neural networks are capable of learning the structure of exact protocols in the $d=2$ case, and could therefore offer a promising route for future investigations in higher dimensions.

## Supplementary Information

Supplementary Information.

## Data Availability

The datasets generated and analyzed during the current study are available in: https://zenodo.org/records/16989647. The code for running the numerical simulations is available in the repository: https://github.com/ManiZart/Prepare-and-measure-and-Entanglement-simulation-beyond-qubits.
