# Supplementary material for: Prepare-and-measure and entanglement simulation beyond qubits
Source: Sci Rep. 2026 May 9;16:21297. doi: 10.1038/s41598-026-51465-3 (PMC13346671; doi:10.1038/s41598-026-51465-3)
Supplement: Supplementary file 1 — Supplementary Information. [file 41598_2026_51465_MOESM1_ESM.pdf]

# Supplementary Material

## Rejection sampling

Rejection sampling[1,2] aims to generate samples from a target distribution $\rho(x)$ using a known distribution $\tilde{\rho}(x)$. The standard procedure is as follows:

- **Rejection sampling**

1. draw a number $u \in [0,1]$, uniformly distributed.
2. Generate a sample $x$ from a known probability density function $\tilde{\rho}(x)$ defined over the domain $\mathscr{D}_X$ of the random variable $X$.
3. choose a constant $M \in [1,\infty)$ satisfying $\rho(x) < M\tilde{\rho}(x)$ for all $x \in \mathscr{D}_X$.
4. Check whether the condition

$$0 < H\left(\frac{\rho(x)}{M\tilde{\rho}(x)} - u\right) \tag{1}$$

is satisfied:

- If yes: accept the sample $x$.
- If not: reject the instance and repeat from 1.

By doing this procedure, the accepted samples will be distributed by the target distribution $\rho(x)$ . The procedure is stochastic, which means there is a nonzero probability that all samples may be rejected. However, it can be shown that the likelihood of this event decreases exponentially with the number of samples[3].

For the rejection method to work properly, the scaling parameter $M$ should be set correctly. However, to do so, one needs to know the proportion of the normalizations of the initial and target distributions, which in our case is not accessible, especially because of the complicated shape of the target distribution $\rho(\Lambda|\mathscr{B})$. To tackle this challenge, we notice that the shared randomness is initially distributed by the Haar measure $\tilde{\rho}(\Lambda) = \mathscr{N}_{Haar}$. So, we introduce a new parameter $M_d := M\mathscr{N}_{Haar}/\mathscr{N}_d$ that casts the comparison step of the rejection sampling algorithm as:

$$0 < H\left(\frac{(\mathrm{Tr}[\Lambda_{i^*}B_{j^*}] - \frac{1}{2})^{\alpha_d} \times H(\mathrm{Tr}[\Lambda_{i^*}B_{j^*}] - \frac{1}{2})}{M_d} - u\right), \tag{2}$$

which doesn't explicitly depend on the normalization factors $\mathscr{N}_d$ and $\mathscr{N}_{Haar}$. However, we should make sure that $M_d$ is set correctly, which implicitly depends on the normalization factors. To address it, we use the numerical trick that if $M_d$ is set appropriately, multiplying it by a constant $\alpha > 1$ will reflect in scaling down the probability of accepting instances by $1/\alpha$. This can be numerically tested by the proportion $N_{\text{out}}/N_{\text{ini}}$. By scaling $M_d$ by $\alpha = 10$, we have found that our protocol, P1, shows the expected statistics of acceptance ratio if we set $M_2 = 0.5$, and $M_3 = M_4 = 0.7$. Besides, for both PRUD-1 and PRUD-2, we have set $M_d = 1$ for all $d$'s.

Let us briefly compare the choice method and the rejection method in PRTQ. In the original version, which uses the choice method, $\vec{\lambda}_0$ and $\vec{\lambda}_1$ are uniformly distributed over $\mathbb{S}^2$. Each $|\vec{y}_b \cdot \vec{\lambda}_0|$ or $|\vec{y}_b \cdot \vec{\lambda}_1|$ can take the role of $u$, as they are independently and uniformly distributed over $[0,1]$. Therefore, during each run, one of these quantities effectively serves as the uniform random variable $u$ in the rejection sampling algorithm, while the other plays the role of the target distribution $\rho(\vec{\lambda}|\vec{y}_b)$. Bob can interchange the roles of $|\vec{y}_b \cdot \vec{\lambda}_0|$ and $|\vec{y}_b \cdot \vec{\lambda}_1|$ ensuring acceptance on every run[4]. In contrast, the standard rejection method is inherently probabilistic; therefore, the communication is only bounded on average. Here, Alice and Bob share only one vector $\vec{\lambda}$, while Bob has an additional local classical resource of a uniformly sampled random number $u \in [0,1]$. In this setup, Bob in step 1 decides whether the sample is accepted or rejected. If rejected, this run of the protocol is aborted. Otherwise, Alice and Bob continue the subsequent steps, and the protocol will produce an outcome. Numerically, these protocols produce the same outcome distributions. However, in the choice method, all samples are accepted, at the cost of using two independent random classical vectors, while in the rejection method, only one vector is required to be shared at the expense of having rejections. For $d = 2$, the probability of acceptance is exactly 1/2 (with $M_2 = 0.5$), meaning that the same number of valid outcomes is obtained on average.

| $d=2$ ; $n=100$ | | | | |
|---|---|---|---|---|
| PM scenario | $N_{\text{ini}}$ $(\times 10^5)$ | $N_{\text{out}}$ $(\times 10^5)$ | $\delta$ $(\times 10^{-2})$ | $std_n/\sqrt{n}$ $(\times 10^{-2})$ |
| P1 | 5.0 | 2.5 | 0.056 | 0.004 |
| PMON-1 | 5.0 | 2.5 | 0.057 | 0.005 |
| PMON-2A | 5.0 | 2.5 | 0.064 | 0.005 |
| PMON-2B | 5.0 | 2.5 | 7.0 | 0.3 |
| PRUD-1 | 20.0 | 2.5 | 0.060 | 0.005 |
| PRUD-2 | 10.0 | 2.5 | 2.3 | 0.1 |
| entanglement scenario | $N_{\text{ini}}$ $(\times 10^5)$ | $N_{\text{out}}$ $(\times 10^5)$ | $\delta$ $(\times 10^{-2})$ | $std_n/\sqrt{n}$ $(\times 10^{-2})$ |
| P1 | 5.0 | 2.5 | 0.101 | 0.005 |
| PMON-1 | 2.5 | 2.5 | 0.092 | 0.005 |
| PMON-2A | 2.5 | 2.5 | 0.133 | 0.007 |
| PMON-2B | 2.5 | 2.5 | 6.9 | 0.3 |
| PRUD-1 | 20.0 | 2.5 | 0.134 | 0.006 |
| PRUD-2 | 10.0 | 2.5 | 2.3 | 0.1 |

**Table 1.** Performance of the protocols for $n=100$ randomized input setups, for $d=2$, for the PM and entanglement scenarios

| $d=3$ ; $n=20$ | | | | |
|---|---|---|---|---|
| $\Delta$ | $N_{\text{ini}}$ $(\times 10^5)$ | $N_{\text{out}}$ $(\times 10^4)$ | $\delta$ $(\times 10^{-2})$ | $std_n/\sqrt{n}$ $(\times 10^{-2})$ |
| 10/24 | 1.5 | 5.9 | 5.6 | 0.3 |
| 11/24 | 2.2 | 6.2 | 2.9 | 0.1 |
| 1/2 | 3.0 | 6.0 | 1.1 | 0.1 |
| 13/24 | 4.2 | 6.1 | 2.2 | 0.3 |
| 14/24 | 6.0 | 6.1 | 4.2 | 0.5 |
| $d=4$ ; $n=20$ | | | | |
| $\Delta$ | $N_{\text{ini}}$ $(\times 10^5)$ | $N_{\text{out}}$ $(\times 10^4)$ | $\delta$ $(\times 10^{-2})$ | $std_n/\sqrt{n}$ $(\times 10^{-2})$ |
| 10/24 | 2.4 | 6.2 | 6.5 | 0.3 |
| 11/24 | 5.0 | 6.3 | 3.1 | 0.2 |
| 1/2 | 9.0 | 6.1 | 2.1 | 0.2 |
| 13/24 | 16 | 6.0 | 3.3 | 0.5 |
| 14/24 | 30 | 5.9 | 5.4 | 0.7 |

**Table 2.** Performance of P1, for $n=20$ randomized input setups as a function of the cutoff $\Delta$ around $\Delta=\frac{1}{2}$, for $d=3$ and $d=4$

## Data tables

The full results of the numerical study are presented in the following tables. As shown in Table 1, for $d=2$, P1, PMON-1, PMON-2A, and PRUD-1 result in accurate predictions as proved theoretically. The ratio $N_{\text{out}}/N_{\text{ini}} < 1$ is a sign of stochasticity for the protocols. Notice that as P1 becomes PRTQ with the rejection method of sampling, the fraction $N_{\text{out}}/N_{\text{ini}} = 1/2$ reflects the probability of acceptance.

In Table 2, the accuracy of the protocol, as a function of cutoff $\Delta$, is provided. It is evident that by deviating from 1/2, the accuracy deteriorates significantly. This provides strong evidence that the exclusion cutoff $\Delta$ should indeed be set to 1/2.

Table 3 depicts results for the randomized inputs of $d=3$, for both scenarios. As seen, P1 achieves the best performance in both cases, with the lowest attained TVD $\delta$.

The $\phi$-parameterized and the CGLMP setups, as the non-random cases, are reported in Tables 4 and 5, respectively. A notable observation is the significant shift in the relative performance of the protocols. In the former case, PMON-2B achieves the lowest error, followed by PRUD-2 and P1, while in contrast, under the randomized setup, PMON-2B ranks as the fourth most accurate protocol.

The CGLMP inequality is a generalization of Bell-type inequalities to higher-dimensional systems and its maximal quantum

| $d=3$ ; $n=100$ | | | | |
|---|---|---|---|---|
| PM scenario | $N_{\text{ini}}$ $(\times 10^5)$ | $N_{\text{out}}$ $(\times 10^5)$ | $\delta$ $(\times 10^{-2})$ | $std_n/\sqrt{n}$ $(\times 10^{-2})$ |
| P1 | 6.0 | 1.2 | 1.13 | 0.06 |
| PMON-1 | 3.6 | 1.2 | 2.09 | 0.09 |
| PMON-2A | 3.6 | 1.2 | 1.18 | 0.06 |
| PMON-2B | 3.6 | 1.2 | 1.62 | 0.07 |
| PRUD-1 | 36 | 1.5 | 1.80 | 0.10 |
| PRUD-2 | 9.0 | 1.1 | 1.46 | 0.05 |
| entanglement scenario | $N_{\text{ini}}$ $(\times 10^5)$ | $N_{\text{out}}$ $(\times 10^5)$ | $\delta$ $(\times 10^{-2})$ | $std_n/\sqrt{n}$ $(\times 10^{-2})$ |
| P1 | 6.0 | 1.2 | 1.11 | 0.03 |
| PMON-1 | 1.2 | 1.2 | 2.00 | 0.04 |
| PMON-2A | 1.2 | 1.2 | 1.14 | 0.04 |
| PMON-2B | 1.2 | 1.2 | 1.54 | 0.03 |
| PRUD-1 | 36 | 1.5 | 1.74 | 0.07 |
| PRUD-2 | 9.0 | 1.1 | 1.49 | 0.03 |

**Table 3.** Performance of the protocols for $n = 100$ randomized input setups, for $d = 3$, for the PM and entanglement scenarios

violation is often used to benchmark nonlocality in higher dimensions. For $d = 3$, it reads

$$\begin{aligned} I_3 := {} & \mathrm{P}(\mathscr{A}_1 = \mathscr{B}_1) + \mathrm{P}(\mathscr{A}_2 = \mathscr{B}_1 - 1) + \mathrm{P}(\mathscr{A}_2 = \mathscr{B}_2) + \mathrm{P}(\mathscr{A}_1 = \mathscr{B}_2) \\ & - \mathrm{P}(\mathscr{A}_1 = \mathscr{B}_1 - 1) - \mathrm{P}(\mathscr{A}_2 = \mathscr{B}_1) + \mathrm{P}(\mathscr{A}_2 = \mathscr{B}_2 - 1) + \mathrm{P}(\mathscr{A}_1 = \mathscr{B}_2 + 1) \leq 2. \end{aligned} \tag{3}$$

Inequality (3) is satisfied for any LHV theory, but can be violated by quantum mechanics, with a maximal value of $I_3 = 2.87$. Here, $\mathrm{P}(\mathscr{A}_i = \mathscr{B}_j + k) := \sum_{k=0}^{2} \mathrm{P}(a, b + k \bmod 3 | \mathscr{A}_i, \mathscr{B}_j)$[5]. We have numerically simulated the behavior of the protocols for the maximum violation input setup. We see that PMON-2A gives the second-worst accuracy, in comparison to the other protocols, with a noticeably larger $\delta$ compared to P1, despite its performance in the randomized case. Additionally, we computed the CGLMP value for each protocol and compared it to quantum value 2.87, as an additional testbed for the numerical performance of the protocols.

| $\phi$-parameterized setup; $d=3$; $n=11$ | | | |
|---|---|---|---|
| PM scenario | $N_{\text{ini}}$ $(\times 10^6)$ | $N_{\text{out}}$ $(\times 10^5)$ | $\delta$ $(\times 10^{-2})$ |
| P1 | 3.0 | 6.0 | 1.50 |
| PMON-1 | 1.8 | 6.0 | 1.72 |
| PMON-2A | 1.8 | 6.0 | 1.55 |
| PMON-2B | 1.8 | 6.0 | 0.87 |
| PRUD-1 | 18 | 7.5 | 2.09 |
| PRUD-2 | 4.5 | 5.6 | 1.10 |

**Table 4.** Performance of the protocols for the $\phi-$parameterized setup with 11 different values of $\phi \in [0, \frac{\pi}{2}]$, for $d = 3$.

Table 6 reports the values for $d = 4$. P1, PMON-2A and PRUD-2, show the highest accuracy with differences of order $\Delta\delta \approx 0.001$, with an overlapping region of $std_n/\sqrt{n} \approx 0.001$. The increase in the $\delta$ from $d = 3$ to $d = 4$ is noticeable among all protocols.

| CGLMP setup; $d = 3$; $n = 4$ | | | | |
|---|---|---|---|---|
| entanglement scenario | $N_{\text{ini}}$ $(\times 10^6)$ | $N_{\text{out}}$ $(\times 10^5)$ | $\delta$ $(\times 10^{-2})$ | CGLMP value |
| P1 | 3.0 | 6.0 | 1.40 | 2.98 |
| PMON-1 | 0.6 | 6.0 | 1.24 | 2.82 |
| PMON-2A | 0.6 | 6.0 | 1.67 | 2.99 |
| PMON-2B | 0.6 | 6.0 | 0.58 | 2.85 |
| PRUD-1 | 18 | 7.5 | 3.37 | 3.10 |
| PRUD-2 | 4.5 | 5.6 | 0.57 | 2.86 |

**Table 5.** Performance of the protocols for the CGLMP setup, for $d = 3$[5].

| $d = 4$ ; $n = 100$ | | | | |
|---|---|---|---|---|
| PM scenario | $N_{\text{ini}}$ $(\times 10^6)$ | $N_{\text{out}}$ $(\times 10^5)$ | $\delta$ $(\times 10^{-2})$ | $std_n/\sqrt{n}$ $(\times 10^{-2})$ |
| P1 | 1.5 | 1.0 | 2.02 | 0.10 |
| PMON-1 | 0.4 | 1.0 | 3.88 | 0.12 |
| PMON-2A | 0.4 | 1.0 | 1.91 | 0.08 |
| PMON-2B | 0.4 | 1.0 | 2.05 | 0.08 |
| PRUD-1 | 7.2 | 1.1 | 3.87 | 0.21 |
| PRUD-2 | 1.5 | 0.9 | 1.96 | 0.10 |
| entanglement scenario | $N_{\text{ini}}$ $(\times 10^6)$ | $N_{\text{out}}$ $(\times 10^5)$ | $\delta$ $(\times 10^{-2})$ | $std_n/\sqrt{n}$ $(\times 10^{-2})$ |
| P1 | 1.5 | 1.0 | 1.81 | 0.05 |
| PMON-1 | 0.1 | 1.0 | 3.85 | 0.04 |
| PMON-2A | 0.1 | 1.0 | 1.77 | 0.04 |
| PMON-2B | 0.1 | 1.0 | 2.04 | 0.03 |
| PRUD-1 | 7.2 | 1.1 | 3.37 | 0.11 |
| PRUD-2 | 1.5 | 0.9 | 1.76 | 0.05 |

**Table 6.** Performance of the protocols for $n = 100$ randomized input setups, for $d = 4$, for the PM and entanglement scenarios

## References


1. Von Neumann, J. *et al.* Various techniques used in connection with random digits. *John von Neumann, Collect. Work.* **5**, 768–770 (1963).
2. Forsythe, G. E. Von neumann's comparison method for random sampling from the normal and other distributions. *Math. Comput.* **26**, 817–826 (1972).
3. Steiner, M. Towards quantifying non-local information transfer: finite-bit non-locality. *Phys. Lett. A* **270**, 239–244 (2000).
4. Degorre, J., Laplante, S. & Roland, J. Simulating quantum correlations as a distributed sampling problem. *Phys. Rev. A* **72**, 062314 (2005).
5. Collins, D., Gisin, N., Linden, N., Massar, S. & Popescu, S. Bell inequalities for arbitrarily high-dimensional systems. *Phys. review letters* **88**, 040404 (2002).
